# SOCS-JAK-STAT inhibitors and SOCS mimetics as treatment options for autoimmune uveitis, psoriasis, lupus, and autoimmune encephalitis

**DOI:** 10.3389/fimmu.2023.1271102

**Published:** 2023-10-26

**Authors:** Rahul Pandey, Marina Bakay, Hakon Hakonarson

**Affiliations:** ^1^ Center for Applied Genomics, Children’s Hospital of Philadelphia, Philadelphia, PA, United States; ^2^ Department of Pediatrics, The University of Pennsylvania School of Medicine, Philadelphia, PA, United States

**Keywords:** JAK-STAT, inflammation, autoimmunity, SOCS (suppressor of cytokine signaling), psoriasis, uveitis, autoimmune encephalitis (AE), lupus

## Abstract

Autoimmune diseases arise from atypical immune responses that attack self-tissue epitopes, and their development is intricately connected to the disruption of the JAK-STAT signaling pathway, where SOCS proteins play crucial roles. Conditions such as autoimmune uveitis, psoriasis, lupus, and autoimmune encephalitis exhibit immune system dysfunctions associated with JAK-STAT signaling dysregulation. Emerging therapeutic strategies utilize JAK-STAT inhibitors and SOCS mimetics to modulate immune responses and alleviate autoimmune manifestations. Although more research and clinical studies are required to assess their effectiveness, safety profiles, and potential for personalized therapeutic approaches in autoimmune conditions, JAK-STAT inhibitors and SOCS mimetics show promise as potential treatment options. This review explores the action, effectiveness, safety profiles, and future prospects of JAK inhibitors and SOCS mimetics as therapeutic agents for psoriasis, autoimmune uveitis, systemic lupus erythematosus, and autoimmune encephalitis. The findings underscore the importance of investigating these targeted therapies to advance treatment options for individuals suffering from autoimmune diseases.

## Introduction

Genetic predisposition, environmental triggers, and dysregulation of the immune system play significant roles in the origin of autoimmune diseases. Additionally, molecular mimicry, epigenetic modifications, hormonal influences, and gut microbiota composition are also relevant factors in the development of these diseases (1, 2). The incidence of these conditions is increasing, affecting around 3% to 5% of people in Western countries (3). Dysregulated cytokine holds a pivotal position in their pathogenesis, making it an attractive target for treatment (4, 5). Cytokines are diverse proteins that mediate cell signaling within the immune system and other host cells, regulating immune responses and inflammation. Cytokines are grouped into various families (6–15), each serving unique functions by binding to specific receptors on target cells and influencing the behavior and function of the immune system. While many are named as interleukins (IL) with numerical identifiers (e.g., IL-2), some, such as TNF-α, IFN-γ, prolactin, and erythropoietin, do not adhere to this naming convention. As integral components of the innate immune system, interferons provide an early defense against infections and contribute to the overall regulation of the immune response (16, 17).

Precise control of cytokine signaling is essential to maintain immune system homeostasis. Monoclonal antibodies targeting specific pathogenic cytokines have transformed autoimmune disease therapy. Nevertheless, there is a necessity for novel therapeutic approaches to tackle relapses and uncontrollable symptoms in affected individuals. Janus Kinase (JAK) inhibitors offer potential as they effectively target crucial cytokines involved in autoimmune and inflammatory diseases (4). This review presents an in-depth examination of the use of JAK inhibitors and SOCS mimetics in treating autoimmune uveitis, psoriasis, systemic lupus erythematosus, and autoimmune encephalitis.

## Janus kinases and signal transducers and activators of transcription

**Figure 1:**
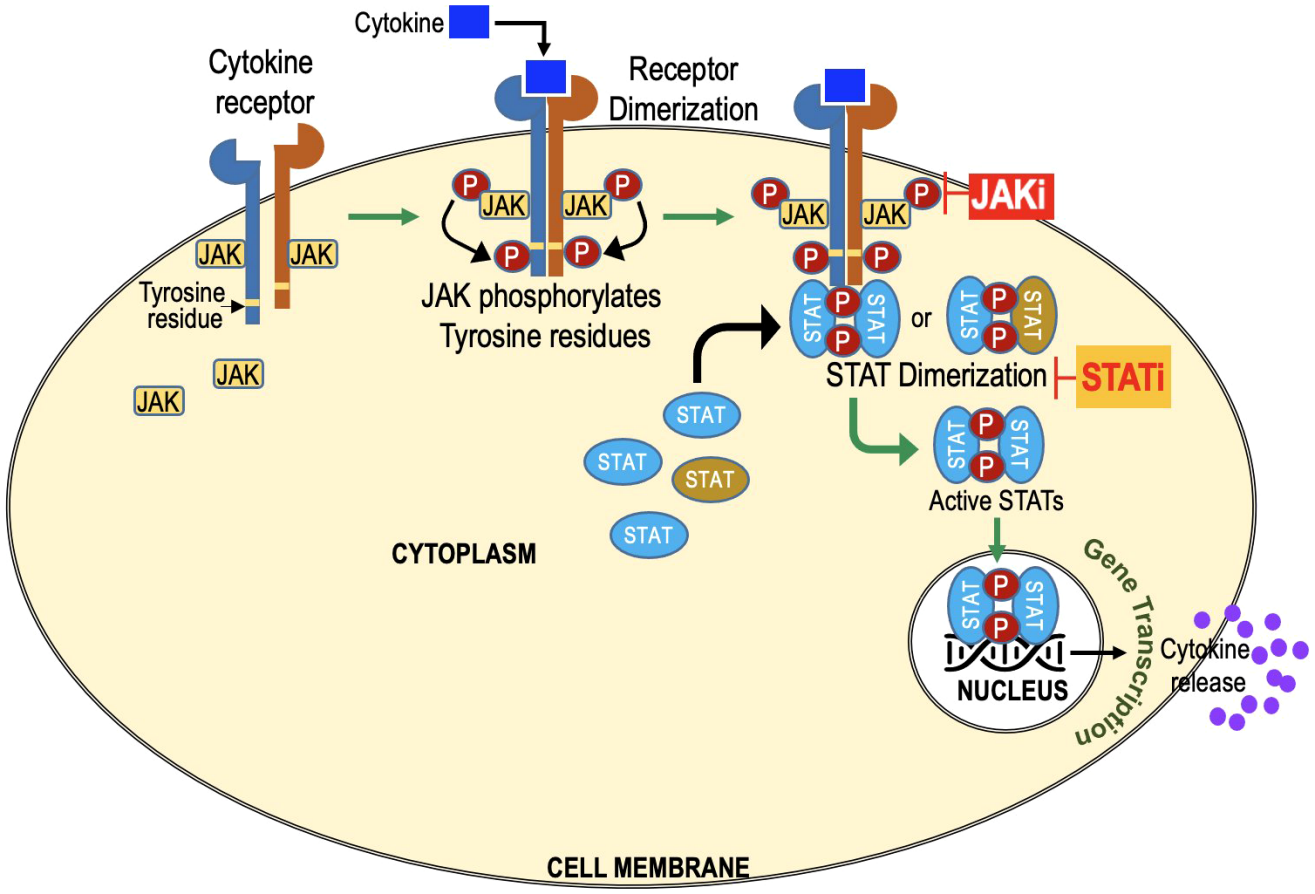
JAK-STAT Signaling Cascade: Key Players in Cellular Regulation and Immune Responses. Extracellular molecules, such as cytokines or growth factors, bind to cell surface receptors, activating Janus Kinases (JAKs). Activated JAKs phosphorylate Signal Transducers and Activators of Transcription (STATs) at specific tyrosine residues, forming homo- or heterodimers in the cytoplasm. STAT dimers then translocate to the cell nucleus and act as transcription factors, regulating gene expression. JAK inhibitors target JAKs. STAT inhibitors target STATs and prevent dimerization and its translocation to nucleus.

The JAK-STAT pathway is a vital signaling cascade that regulates diverse biological processes, including immune responses, cell growth, and differentiation. It is named after its key components, Janus Kinases (JAKs), which were discovered 30 years ago. The term “Janus” originates from Roman mythology, symbolizing transitions, and duality. JAKs possess two domains: a kinase domain responsible for phosphorylation and a pseudokinase domain acting as a negative regulator, giving them the name “Janus Kinases.” The term “Signal Transducers and Activators of Transcription” describes the primary function of these proteins in the JAK-STAT pathway. Once JAKs phosphorylate STATs, the activated STATs act as signal transducers by relaying the extracellular signal from the cell surface receptors to the cell nucleus. Once in the nucleus, STATs function as transcription factors, activating the transcription (gene expression) of specific target genes (**Figure 1**).

**Figure 2:**
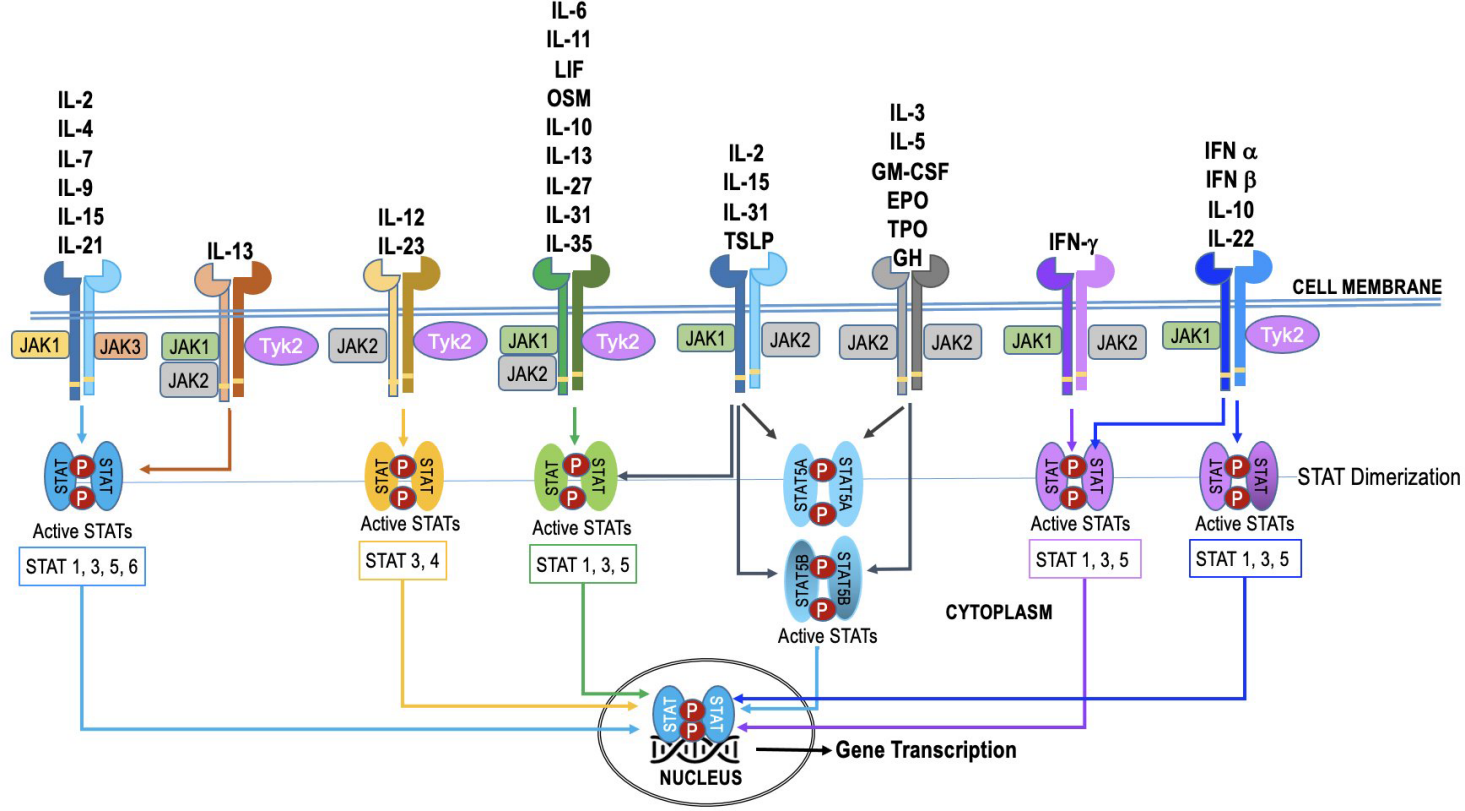
The specificity and redundancy of the JAK-STAT pathway. Different JAK family members are linked to specific cytokine receptors, and they trigger distinct STAT proteins within the pathway. This selective activation allows for fine-tuning of cellular responses to different stimuli. However, the JAK-STAT pathway also demonstrates redundancy, especially in certain cellular contexts. This redundancy ensures that essential functions are maintained even if one JAK-STAT axis is compromised or inhibited. For instance, various cytokines can activate both JAK1 and JAK2, leading to phosphorylation of STAT1 and STAT3, respectively. This redundancy allows cells to respond to multiple cytokines and ensures a robust and adaptable immune response.

The JAK family in humans consists of four members: JAK1, JAK2, JAK3, and TYK2. These four JAK proteins are utilized by over 50 cytokines, leading to substantial overlap in their usage (**Figure 2**). The STAT family in humans consists of seven members - STAT1, STAT2, STAT3, STAT4, STAT5A, STAT5B, and STAT6. Additionally, some cytokines and chemokines signal through mechanisms independent of JAK-STAT (18). The JAKs and STATs are differentially expressed in various cell types, and their activation can lead to distinct downstream effects (4, 19–24). Selective activation of this pathway enables precise adjustment of cellular reactions to diverse triggers. Nevertheless, in specific cellular scenarios (as illustrated in **Figure 2**), the JAK-STAT pathway exhibits redundancy. This redundancy guarantees the preservation of vital functions, even when one JAK-STAT axis encounters disruption or inhibition. Such redundancy empowers cells to react to numerous cytokines, ensuring a resilient and flexible immune response.

The effective functioning of the immune system depends heavily on the JAK-STAT signaling pathway, and disruptions in cytokine-JAK-STAT signaling can result in immunodeficiency (25, 26). If the JAK1 or JAK2 genes are deactivated in the germline, the fetus in non-viable (27, 28). Certain primary immunodeficiencies result from genetic mutations affecting JAK-STAT signaling components, leading to immunological abnormalities and increased susceptibility to infections (29–36). Furthermore, mutations impairing the function of STAT proteins can cause both immunodeficiency and autoimmunity (37–39). The occurrence of autoimmunity is believed to be associated with the disruption of regulatory communication between STAT proteins (40, 41). Job Syndrome (Hyper-IgE Syndrome), a rare genetic disorder caused by impaired functioning of STAT3, leads to compromised JAK-STAT signaling (42, 43). Overall, the loss of cytokine-JAK-STAT signaling interferes with critical immune functions, compromising the development, differentiation, and response of immune cells, as well as the regulation of immune mediators and inflammatory processes. Overactivation of cytokine signaling can have detrimental effects too (25, 26, 44). The overactive JAK-STAT pathway promotes production of pro-inflammatory cytokines and activation of immune cells and contributes to the development of autoimmune diseases. Similarly, in cancer, dysregulated JAK-STAT signaling can play a role in promoting uncontrolled cell growth and survival (4, 45–47).

## The SOCS protein family

The Suppressor of Cytokine Signaling (SOCS) protein family is a group of proteins that play a vital role in regulating and inhibiting cytokine signaling pathways. These proteins act as negative feedback regulators, dampening excessive immune responses and maintaining immune system balance. SOCS proteins achieve this by binding to specific signaling proteins, including Janus kinases and cytokine receptors, and interfering with their activity, ultimately controlling the duration and intensity of immune and inflammatory reactions. The SOCS protein family consists of numerous members, namely SOCS1, SOCS2, SOCS3, SOCS4, SOCS5, SOCS6, SOCS7, and CIS (Cytokine-Inducible SH2-Containing protein), with CIS being the initial SOCS member to be discovered (48). Since their discovery in 1997 simultaneously by three groups (48–50), these have gained widespread recognition due to their prominent role in the negative modulation of signaling pathways subsequent to cytokine engagement with the receptor complex. In the late 1990s, researchers were investigating how cells modulate their responses to cytokines to prevent excessive inflammation and maintain immune system balance. In this regard, retroviral expression screen was developed to investigate how cells modulate their responses to cytokines. During this screen, an identified cDNA sequence encoded a compact protein featuring an SH2 domain, displaying resemblance to the cytokine-inducible SH2-containing (CIS) protein (48). This specific cDNA was designated as SOCS1 and subsequently played a pivotal role in the revelation and cloning of an additional six SOCS family members (SOCS2, 3, 4, 5, 6 and 7). Comparable to CIS, it was observed that SOCS1, SOCS2, and SOCS3 were responsive to cytokine stimulation (50, 51). Studies utilizing a combination of molecular biology techniques, gene expression profiling, and cell culture experiments collectively provided strong evidence that SOCS1 is not solely triggered by cytokine activation but also functions as a standard negative-feedback modulator, effectively limiting JAK signaling (52). The discovery of SOCS1 shed light on a crucial aspect of immune system regulation and paved the way for further research into the broader implications of SOCS proteins in various physiological and pathological contexts. Notably, within autoimmune disorders, multiple SOCS proteins (SOCS1, SOCS3, SOCS5, and CIS) function as potent negative regulators significantly contribute to the underlying mechanisms driving the diseases’ progression. SOCS1 helps to control inflammation, immune responses, and cell differentiation in various autoimmune diseases. SOCS3 targets diverse cytokine pathways by binding to receptors and JAKs and curbs signal transmission, moderating inflammation and immune responses in type 1 diabetes, inflammatory bowel disease (IBD), and psoriasis. CIS competes with STAT proteins for cytokine receptor binding. It fine-tunes cytokine responses and prevents uncontrolled immune reactions. SOCS4-7 extend their functions beyond cytokine signaling, with notable roles in regulating receptor tyrosine kinases that mediate hormonal effects like insulin and growth factors such as epidermal growth factor (EGF) (24, 53). SOCS5 dampens cytokine signaling by interacting with and inhibiting JAKs. While its precise role in autoimmune disorders remains unclear, emerging evidence suggests its potential involvement in autoimmune uveitis (54), type 1 diabetes (55), multiple sclerosis (56), SLE (57) and EAE (58). SOCS-6 was revealed as a suppressor of p56(lck) in yeast two-hybrid screening. By promoting ubiquitin-dependent proteolysis, SOCS-6 acts as a negative regulator of T cell activation (59). In contrast, SOCS7^−/−^ mice exhibited varying immune-related characteristics contingent on their genetic makeup. Precise role of SOCS6 and SOCS7 in the intricate landscape of immune regulation is still emerging (60–62). Although CISH and SOCS1–3 hold evident prominence in the context of the immune system and extensively reviewed in diseases context elsewhere (44, 63), recent investigations suggest that SOCS4–7 might also play a role, underscoring the need for further exploration into these proteins. Although the exact roles they play in distinct autoimmune conditions might necessitate further scrutiny, their significance is progressively acknowledged, offering possibilities for upcoming therapeutic strategies directed at modulating these regulatory pathways.

## Structure of SOCS proteins

**Figure 3:**
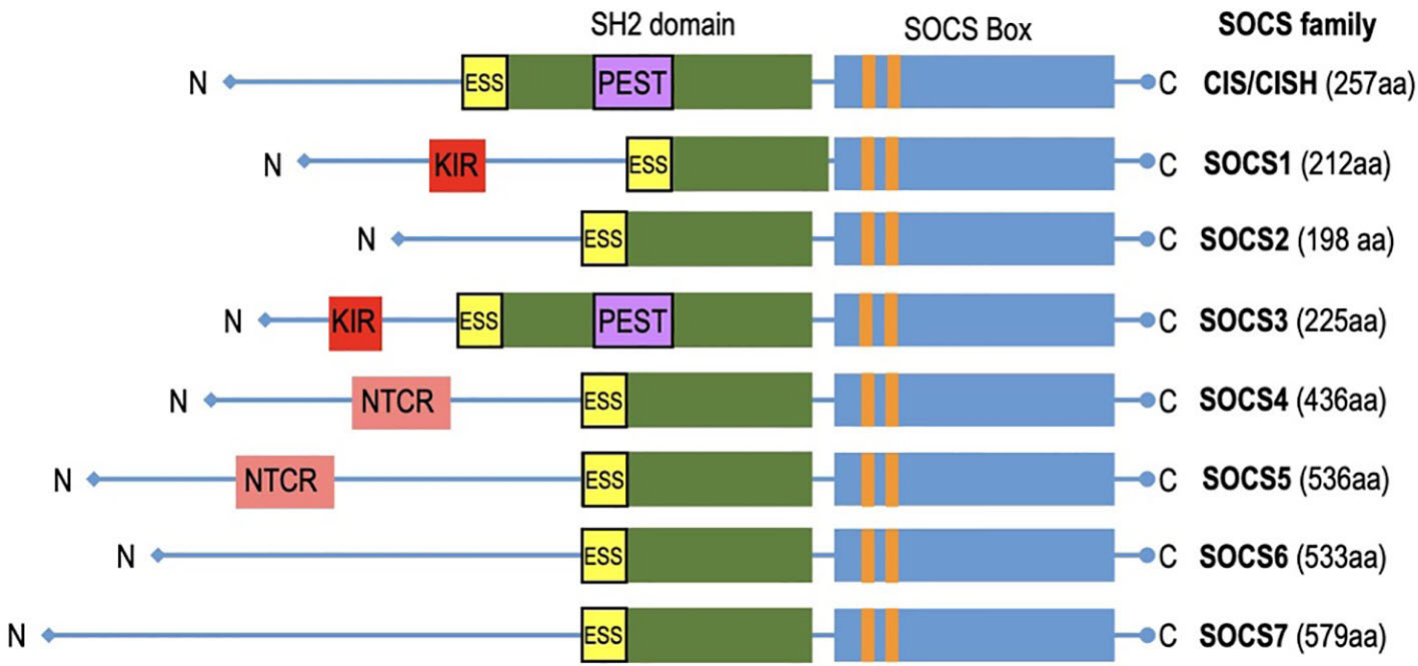
The diagram depicts CIS/SOCS family proteins featuring conserved SOCS box in all of them. Notably, SOCS1 and SOCS3 exhibit unique kinase inhibitory region (KIR) which acts as a pseudo substrate.

SOCS (Suppressors of Cytokine Signaling) proteins exhibit a structured composition that encompasses several key elements essential for their regulatory functions (**Figure 3**). A fundamental feature found in all SOCS family members is the SOCS box, a conserved domain pivotal in protein-protein interactions, especially with components of the ubiquitin ligase complex. SOCS proteins interact with phosphorylated tyrosine residues on target substrates through their SH2 domain (64). The SH2 domains of SOCS proteins possess an additional N-terminal α-helical extension called the extended SH2 domain (ESS) (65). The SOCS controls assembly of E3 ubiquitin ligase complex and contains two motifs: the Elongin B/C (BC) box and the Cullin (Cul) box (66, 67). The N-terminal varies significantly (68). The N-terminus of SOCS4-7 are notably longer (69). Specific motifs within the N-terminal domains have been identified in related SOCS proteins. Notably, SOCS1 and SOCS3 feature a unique kinase inhibitory region (KIR) that binds and inhibits JAKs (70), whereas SOCS3 and CISH contain a PEST motif located between the SH2 domain and SOCS box (48, 65). SOCS4 and SOCS5 have distinct N-terminal conserved region (NTCR) with unknown function (69). SOCS proteins are vital in immune coordination, making them potential targets for therapeutic intervention. The evolutionary relationship between SOCS proteins is reflected in their similarity. This conservation is seen in humans and other mammalian species, which possess equivalent sets of SOCS proteins. Higher vertebrates have homologs for each SOCS protein, and teleost fish have additional duplicates. Recent discoveries have revealed intriguing differences among these proteins, emphasizing their importance in health and disease. SOCS proteins are promptly induced upon cytokine receptor signaling but are rapidly degraded when signaling subsides, remaining inactive in quiescent cells acting as negative feedback regulators. This unique structural arrangement allows SOCS proteins to exert regulation over cytokine signaling pathways, facilitating functions such as competitive binding, targeted protein degradation, and the inhibition of kinase activity. In doing so, SOCS proteins contribute to the maintenance of immune system homeostasis.

## Mechanisms of SOCS action

**Figure 4:**
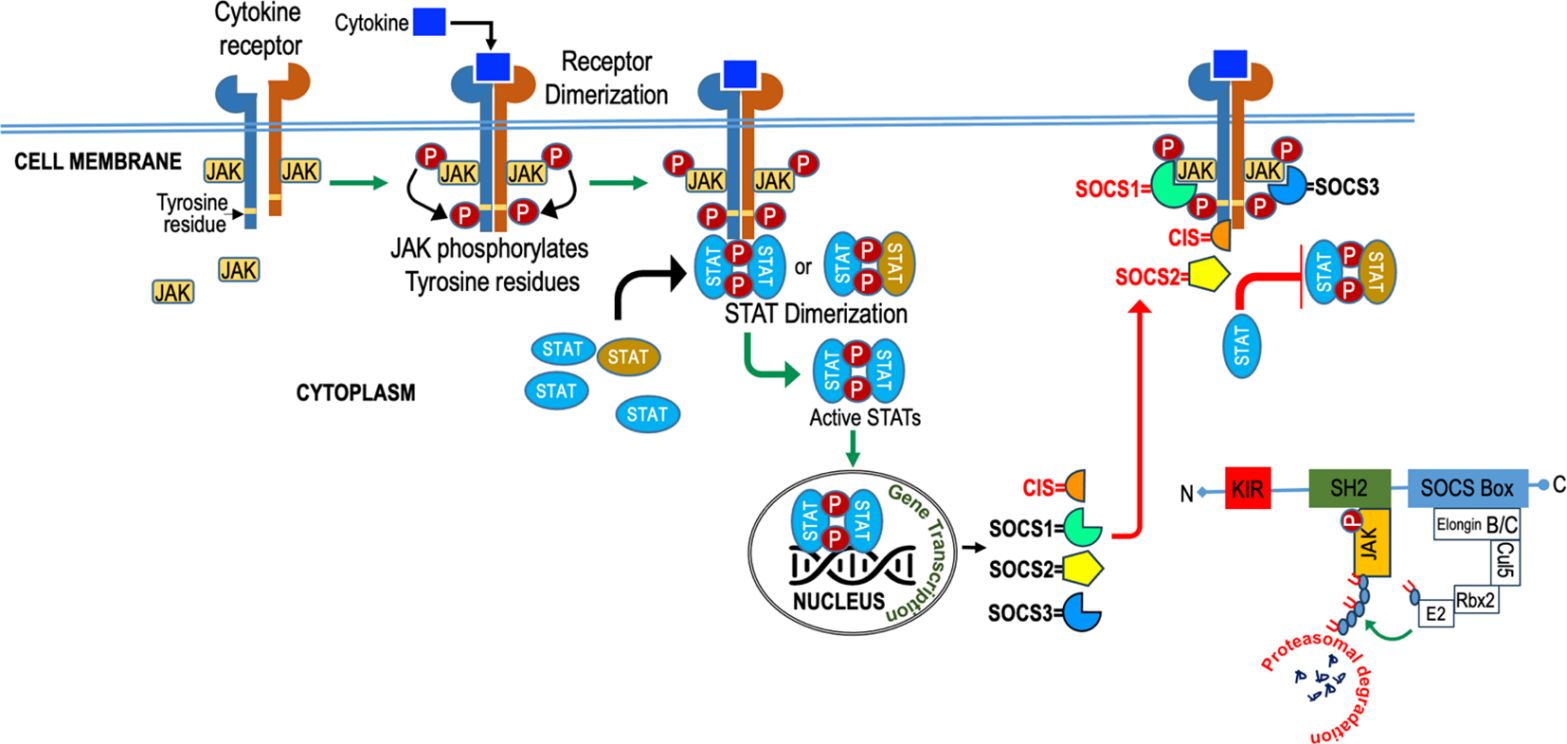
Diagram illustrating the role of SOCS (Suppressor of Cytokine Signaling) in cytokine signaling. SOCS molecules act as crucial regulators, inhibiting excessive cytokine responses to maintain balanced immune function.

SOCS employ diverse mechanisms, as illustrated in **Figure 4**, to regulate cytokine signaling, encompassing competitive binding, protein degradation or re-routing, and inhibition of kinase activity. These strategies rely on the presence of specific protein domains and motifs in varying combinations to tightly regulate cytokine signaling and maintain immune system homeostasis. The SH2 domain of SOCS proteins, reviewed elsewhere [24, 92], usually bind to the cytokine receptor signaling complex or downstream signaling proteins by interacting with appropriate motifs containing phosphotyrosine residues (24, 71). Competitive binding hinders the docking of STAT and other proteins, effectively suppressing their subsequent activation through steric interference (24). SOCS proteins degrade target proteins by interacting through their SH2 domain (72, 73) with phosphorylated JAK proteins or receptors, assembling the E3 ubiquitin ligase complex, which transfers ubiquitin to target substrates for degradation (74) and potential re-routing of associated proteins (75). SOCS1 binds to JAKs, while SOCS3 binds to receptors (76, 77), and they both directly inhibit JAK kinase activity by blocking the substrate-binding groove of the JAK kinase domain, acting as a pseudo substrate (78, 79). SOCS1 has a unique nuclear localization signal (NLS) and interacts exclusively with p65 in the nucleus. This interaction effectively curtails prolonged p65 signaling and halts the expression of NF-kB-inducible gene (80–83). SOCS proteins also regulate cytokine-responsive genes by interacting with transcription factors or chromatin modifiers (83).

At the post-transcriptional level microRNA-155 has been identified as a natural regulator of SOCS1 mRNA (84). miRNA-155 dysregulation has been associated with Inflammation (85–87), heart failure (85), neurodegenerative diseases (88, 89), antiviral immune response in SARS-CoV2 infection (90) and cancers (91–95). Additionally, SOCS1 is regulated post-translationally by several kinases like v-abl, pim1, and pim2. Phosphorylation by these kinases blocks the SOCS Box from binding to Elongin C, a key step in recruiting the E3 complex (96, 97). Numerous cytokines and growth factors stimulate SOCS1, implying its role in moderating and buffering the cellular and inflammatory responses initiated by these molecules suggesting that SOCS1 plays a role in downregulating/buffering the cellular/inflammatory responses triggered by these cytokines (98–102).

## SOCS1 in autoimmunity

**Figure 5:**
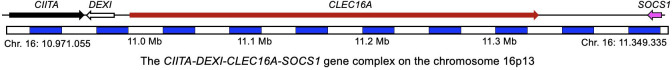
Schematic outline representing the genetic region on chromosome 16p13, encompassing *CIITA-DEXI-CLEC16A-SOCS1*. The genomic coordinates are sourced from Genome Reference Consortium Human Build 38.

The *SOCS1* gene is located on chromosome 16 alongside *CLEC16A* (cytogenetic location: 16p13.13; genomic coordinates [GRCh38]: 16:11,254,417-11,256,204). This region, around 530 kb on chromosome 16p13.13, harbors four genes (*CIITA-DEXI-CLEC16A-SOCS1*) (**Figure 5**).

In 2007, we initially identified a region on chromosome 16p13 mapping to KIAA0350, now known as C-type lectin-like domain family 16A (*CLEC16A*), as a novel susceptibility locus for type 1 diabetes (T1D) (103). *CLEC16A* is situated between two neighboring genes: *CIITA*, crucial for MHC Class II expression, and *SOCS1*, a negative regulator of cytokine signaling and *DEXI.* The susceptibility sites, *CIITA-DEXI-CLEC16A-SOCS1*, are common across various autoimmune conditions (104–108). *SOCS1* and *CIITA* are recognized contributors to inflammation and autoimmunity (106, 107, 109–111). SOCS1 plays vital role in immune cell homeostasis and modulating inflammation through its intricate modulation of cytokine signaling (63, 112). Variations in *SOCS1*’s 5’ UTR (rs243324 and rs441349) have been identified as susceptibility factors for multiple sclerosis (MS) in cytokine pathway screenings (105, 113). The *CLEC16A* locus, encompassing *SOCS1*, is now associated with 21 autoimmune diseases. These include type 1 diabetes (103, 114–126), multiple sclerosis (MS) (104, 105, 119, 125, 127–140), primary adrenal insufficiency (PAI) (141, 142), systemic lupus erythematosus (SLE) (143–146) Crohn’s disease (CD) (147), selective immunoglobulin A deficiency (IgA) (148), alopecia areata (AA) (149, 150), juvenile idiopathic arthritis (JIA) (151), rheumatoid arthritis (RA) (119, 151), primary biliary cirrhosis (PBC) (152–155), asthma (156–162), Crohn’s disease (CD) (147), allergic rhinitis (AR) (163, 164), autoimmune thyroid diseases (ATD) (115, 165), common variable immunodeficiency (CVID) (166), eosinophilic esophagitis (EE) (167), juvenile idiopathic arthritis (JIA) (151), Selective IgA deficiency (148), Celiac disease (168), systemic sclerosis (169) and even Parkinson’s Disease (PD) (170, 171) as reviewed (1).

The field of autoimmune research is constantly progressing, and over the past three years there has been a major advancement and shift to identify new therapeutic pathways for autoimmunity sufferers. 2020, study highlighted the importance of restoring immune homeostasis and tolerance, with a particular emphasis on therapies aimed at promoting, activating, or delivering regulatory T cells (Tregs). These approaches have shown promise in the pursuit of curing or effectively managing autoimmune diseases (172). 2021, another study discussed the safety and effectiveness and challenges associated with mesenchymal stem cell (MSC) treatment for people with autoimmune liver disease (173). In 2021, a study highlighted the role of patients’ microbiomes in the management of systemic sclerosis and immunoglobulin G4-related disease (IgG4-RD) (174). Recently, we reported an autoimmune and lipodystrophic phenotype using a mouse model, *Clec16a* ^ΔUBC^ *(* 175). This study revealed a link between CLEC16A, lipid metabolism, and immune disruptions. Treating *Clec16a* ^ΔUBC^ mice with the tofacitinib, partly alleviates the lipodystrophic issue and enhances survival. Tofacitinib affects autophagy and JAK-STAT mediated SOCS signaling (175). The *CLEC16A* locus role in autophagy (176, 177), mitophagy (178), immune regulation and neurodegeneration (179, 180), makes it a promising target in autoimmune disorders. Genetic interactions and environmental triggers contribute to immune dysregulation, resulting in inflammation, autophagy, and cell death in autoimmune disorders. Exploring the intricate crosstalk and potential synergy between SOCS-mediated cytokine regulation and the contributions of CLEC16A to autoimmune pathogenesis could unveil novel insights into disease mechanisms. Investigating the convergence of *SOCS* and *CLEC16A* in the context of autoimmunity might provide a comprehensive understanding of the complex interplay between regulatory and predisposing factors, offering opportunities for innovative treatments.

## SOCS-mimetics

The discovery of the SOCS1-KIR binding site on JAK2 has led to the development of SOCS1 mimetics and antagonists with potential immune response enhancement capabilities (181). Specifically, SOCS1-KIR, a unique mimetic peptide, consists solely of the KIR domain, acting as a pseudosubstrate for JAK1, JAK2, and TYK2, but not interacting with JAK3 (78). Several Jakinibs have received FDA approval for specific autoimmune/inflammatory disorders and are currently being evaluated for additional conditions. A significant advantage of SOCS1-KIR as a therapeutic candidate is its structural similarity to natural SOCS1 protein. Nonetheless, mimetic peptide drugs present drawbacks encompassing potential higher costs, restricted permeability, proteolytic vulnerability, short half-life, swift *in-vivo* clearance, and limited oral bioavailability. To address these limitations, various strategies are currently being employed to improve the properties of peptide drugs (182).

Over the past decade, different types of SOCS mimetics and antagonists have surfaced and been subject to testing. For instance, Tkip (mimetic of SOCS1, WLVFFVIFYFFR) (183, 184) is based on the SOCS-KIR domain. The mimetic peptide showed promising results similar to naturally occurring SOCS1, reduced the inflammatory phenotype in murine encephalomyelitis model (EAE). Tkip inhibits IFN-γ signaling and suppressed the effector functions of T-cells. It compensates for low levels of endogenous SOCS1 and SOCS3 associated with EAE (185). Topical administration of SOCS1-KIR peptide was shown to successfully prevent uveitis and ocular damage (186). Furthermore, a different mimetic peptide, R9-SOCS1-KIR, successfully suppressed autoimmune uveitis EAU in mice by inhibiting the cations of IFN-γ, TNF-α, and IL-17, consequently preventing ocular pathology (187).

Additionally, cell-penetrating forms of SOCS1 (CP-SOCS1) and SOCS3 (CP-SOCS3) have also been developed and tested in various disease models. Controlled, intracellular delivery of recombinant CP-SOCS1 has been shown to suppress the IFN-γ signaling (188). It interacts similar to endogenous SOCS1, and the extent of inhibition depended on the dosage. Another, CP-SOCS3 peptide was developed and tested to treat acute liver injury driven by LPS in mice. It effectively suppressed the inflammation driven by TNF-α and IFN-γ. A previous *in-vitro* study demonstrated that CP-SOCS3 exhibited similar actions to endogenous SOCS3 (189). Deletion of the SOCS box domain in both CP-SOCS1 and CP-SOCS3 in the mimetic peptide is a strategic modification that yields significant benefits in terms of both activity and longevity. The SOCS box domain is a crucial part of the natural SOCS-1 protein, serving as a recognition site for ubiquitin ligases that lead to the degradation of the protein. By removing this domain, the modified CP-SOCS1 and CP-SOCS3 peptides gain an advantage in their intracellular presence, as they are no longer subject to rapid degradation, ensuring their persistence within the cellular environment to inhibit pro-inflammatory signaling pathways over an extended period. By extending their activity and increasing their potency, the modified peptides provide a more robust and durable anti-inflammatory effect, making them promising candidates for therapeutic interventions aimed at curbing excessive immune responses and managing inflammatory conditions (187–189).

**Figure 6:**
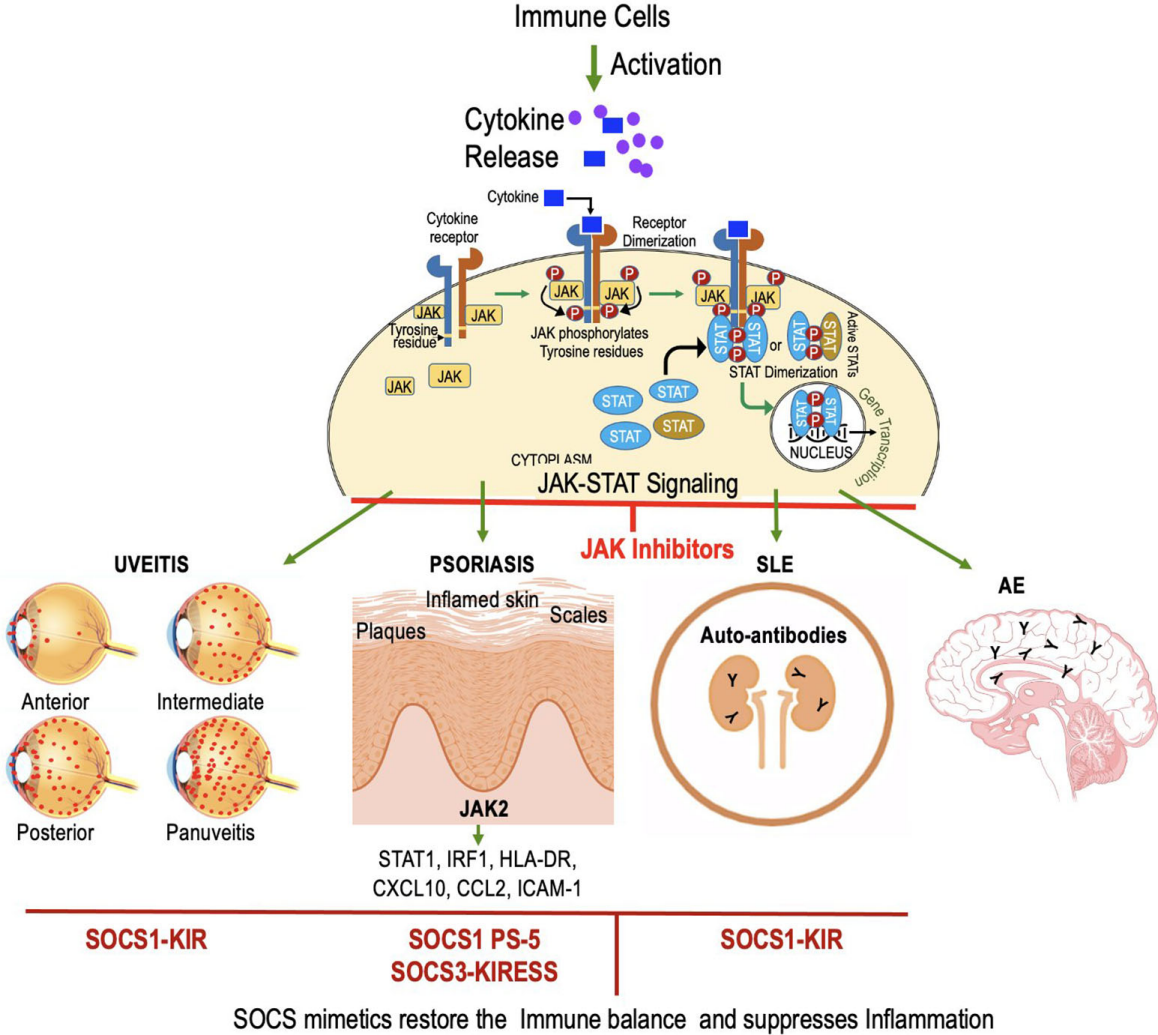
JAK inhibitors and mimetics show promise in treating autoimmune disorders like uveitis, psoriasis, SLE, and AE. SOCS1-KIR peptide mimics SOCS1 to inhibit pro-inflammatory cytokines, reducing uveitis. In psoriasis, characterized by the infiltration of immune cells and excessive keratinocyte proliferation, the action of SOCS1 and the PS-5 peptide involves the inhibition of pro-inflammatory molecules through the targeting of JAK2. In SLE, SOCS1-KIR treatment alters lymphocyte phenotype, potentially mitigating SLE pathology. SOCS1 mimetic has not been tested for AE.

The potential of manipulating SOCS proteins as a therapeutic strategy for immune-related disorders is underscored by these findings. Among the SOCS peptides, SOCS1-KIR stands out as a promising mimetic with unique interactions and potential therapeutic benefits. It holds great promise as an addition to the arsenal of Jakinibs and it is our belief that the development of peptide drugs will persist in the future, offering new avenues for therapeutic interventions with notably fewer adverse events. While peptide drugs face certain limitations, ongoing research and technological advancements offer opportunities to improve their efficacy and overcome challenges related to their absorption, distribution, metabolism, and excretion characteristics. The restoration or enhancement of SOCS1 function is proposed to suppress excessive immune responses in autoimmune and autoinflammatory conditions (71, 108, 190). **Table 1** and **Figure 6** provide an overview of the application of SOCS mimetics in the therapeutic management of autoimmune uveitis, lupus, and psoriasis. SOCS mimetics have not yet undergone testing for autoimmune encephalitis. However, treatment options for other autoimmune and inflammatory disorders using mimetic peptides can be found in separate literature due to space constrain.

**Table 1:** SOCS mimetic peptides in autoimmune uveitis, lupus, and psoriasis.

Peptide	Sequence	Uveitis	Lupus	AE	Psoriasis
Tkip	WLVFFVIFYFFR				
SOCS1-KIR	^53^DTHFRTFRSHSDYRRI^68^	(186, 187, 191, 192)	(193)		
PS-5	DTC(Acm)RQTFRSH				(194)
KIRESS-SOCS3	^22^LKTFSSKSEYQLVVNAVRKLQESG^45^				(195, 196)

## Autoimmune uveitis

Uveitis refers to inflammation in any part of the uveal tract. Uveitis can stem from localized eye issues or systemic disorders affecting the body like autoimmune conditions (e.g., Bechet syndrome, ankylosing spondylitis) or infections (e.g., tuberculosis, herpes) (197–199). Uveitis can affect a single or multiple uveal structures of the eye and may also involve adjacent ocular tissues. Injuries to the eye, certain medications, and environmental factors play roles. Uveitis is a significant cause of ocular morbidity, leading to legal blindness in developed societies, with prevalence influenced by geography, ethnicity, and unknown factors in 30-60% of patients. Autoimmune or autoinflammatory uveitis is prevalent in developed countries, with different forms based on the location of inflammation within the uveal tract (anterior, intermediate, posterior, or panuveitis). The pathogenesis involves complex interactions between genetic, environmental, and immunological factors, and prompt diagnosis and treatment are crucial to prevent severe vision impairment or blindness (200).

## EAU pathogenesis

Experimental autoimmune uveitis (EAU) is a valuable model for studying the pathogenesis of human autoimmune uveitis (192, 197, 199, 201–203). EAU is primarily triggered by a dysregulated immune response directed against ocular antigens, specifically retinal antigens. Antigen-presenting cells, such as dendritic cells, capture retinal antigens and present them to autoreactive T cells in the draining lymph nodes. This interaction leads to the activation of pathogenic T cell subsets, particularly Th1 and Th17 cells, which migrate back to the eye, triggering an inflammatory cascade and attract secondary effectors (204–206). In EAU models, genetic factors influence the immune response (207). Human leucocyte antigens (HLA) association with different forms of uveitis have been identified since the early 1970s (208). Recent progress in genetics of Uveitis has shown novel associations of AAU, BD and BSCR with HLA-B27, HLA-B51, and HLA-A29, respectively (207). Inflammation within the eye involves the recruitment of various immune cells, including neutrophils, macrophages, and T cells, leading to tissue damage, breakdown of the blood-retinal barrier, and retinal destruction. The release of pro-inflammatory cytokines, chemokines, and growth factors further amplifies the immune response, leading to the characteristic clinical features of uveitis (209–213). The intricate interplay between immune cells, cytokines, and ocular tissues in EAU provides valuable insights into the complex pathogenesis of autoimmune uveitis in humans, guiding the development of targeted therapies to mitigate the detrimental effects of the disease. The integration of human studies, equine models, and mouse uveitis models highlights Th17 cells and the JAK-STAT pathway’s significance in uveitis pathogenesis, with SOCS molecules playing crucial roles (192, 213).

## Treatment of autoimmune uveitis

The JAK-STAT pathway regulates uveitis inflammation. Uveitis treatment can be categorized into local therapy, systemic therapy, or a combination of both. STAT3 inhibitors like ORLL-NIH001, blocking lymphocyte entry into the retina, shows promise for uveitis treatment (214).

Novel approaches to uveitis treatment aim to improve outcomes and mitigate its effects. These therapies include targeting cytokines and cytokine receptors, Janus kinases, the STAT3 pathway (via synthetic inhibitors), SOCS mimetic peptide therapy, and therapeutic cytokines like IL-35. Employing immunosuppressive cytokines such as IL-27 and IL-35, as well as using small molecule inhibitors to counteract proinflammatory cytokines, are noteworthy therapeutic strategies. For instance, ustekinumab specifically targets IL-12 and IL-23, while anakinra focuses on IL-1. IL-35 treatment was protective in mice with EAU. Conversely, mice lacking IL-35 (p35 knockout, or KO, mice) or with impaired IL-35 signaling (IL-12Rβ2 KO mice) experienced severe uveitis (215). Additionally, IL-35 exosomes effectively combat instability and short lifespan concerns, providing protection against severe uveitis in mice (216). These findings suggest role of IL-35 in modulating Uveitis through cytokine therapy. In mice, the absence of STAT3 within the CD19 B cell compartment (known as CD19-STAT3KO mice) worsens uveitis EAU (217). Another aspect influenced by STAT3 is miR-155-5p (miR-155), which is linked to ocular inflammation. STAT3 activates miR-155, and this interplay between STAT3 and miR-155 contributes to severe uveitis by fostering the expansion of pathogenic Th17 cells (218, 219). This suggests STAT3 pathway employed by Th17 cells is a potential strategy for uveitis.

Currently, TNF-α inhibitors are the main biologic agents used (220). Uveitis clinical trials of JAK inhibitors to date are listed in **Table 2**. The search for a potentially innovative uveitis therapeutic option continues. There are three clinical uveitis trials with JAK-STAT inhibitors now underway: Brepocitinib (NCT05523765) and Baricitinib (NCT04088409 and NCT05651880).

**Table 2:** Uveitis clinical trials of JAK inhibitors, chronological by the start date.

Intervention	Specificity of Inhibition	Conditions	NCT Number	Phases	Study Status	Enrollment	Start Date (M/D/Y)	Completion Date (M/D/Y)
Filgotinib & Prednisone	JAK1	Noninfectious Uveitis	NCT03207815	2	Terminated	74	7/26/2017	04/22/2021
Tofacitinib	JAK3>JAK1>>(JAK2)	Uveitis Scleritis	NCT03580343	2	Unknown	5	4/4/2019	04/04/2021
Baricitinib & Adalimumab	JAK1/JAK2	Chronic Anterior Uveitis	NCT04088409	3	Active, Not Recruiting	40	10/16/2019	07/15/2023
Brepocitinib (PF-0670841)	JAK1/TYK2	Noninfectious Uveitis	NCT05523765	2	Recruiting	24	11/14/2022	07/2024
Baricitinib	JAK1/JAK2	Active Non-anterior Non-infectious Uveitis	NCT05651880	3	Not Yet recruiting	33	2/23/2023	08/01/2026

## SOCS1 mimetics in autoimmune uveitis

The JAK-STAT pathway is a crucial regulator in NIU, and SOCS1 mimetics are promising to address SOCS1 deficiency. The SOCS1-KIR peptide (53DTHFRTFRSHSDYRRI68) is a noteworthy candidate due to its similarity to natural SOCS1 and its ability to inhibit JAK1/2 and TYK2, extending survival in SOCS1-deficient mice (183, 184, 221, 222). SOCS1 expression in the retina reduces inflammation and lymphocyte recruitment (223). Locally administered SOCS1-KIR peptide shows positive effects in mitigating disease symptoms in rodents (186, 187, 191).

A pilot study recently examined the safety and effectiveness of the SOCS1 mimetic peptide for treating equine recurrent uveitis (ERU), which serves as a model for human recurrent uveitis (RU) (192). RU is a debilitating autoimmune disease that can cause visual impairment in both horses and humans, despite existing treatments. Cases unresponsive to current treatments remain a significant concern. In ERU, inflammatory process driven by cytokines utilizes JAK2 signaling and contributes to blindness. SOCS1, which limits JAK2 activation, is often deficient in autoimmune disease patients. Bioinformatics and binding assays confirmed the SOCS1-KIR peptide’s potential to bind to equine JAK2. The safety of SOCS1-KIR peptide in equine eyes was initially assessed in a pilot study using healthy horses without equine recurrent uveitis. Two experimental horses received topical administration of SOCS1-KIR or a carrier for two weeks, while two other horses received intravitreal injection of SOCS1-KIR. Ophthalmic, physical exams, and electroretinography were conducted, showing that both topical and intravitreal SOCS1-KIR were safe for equine eyes (192). The results suggest that topical SOCS1-KIR treatment may restore immune tolerance in the eye and inhibit antigen presentation, a factor in triggering inflammation. Further testing in human and equine samples is essential for a comprehensive understanding of the mechanisms of action. These findings strongly encourage additional exploration of the mechanistic aspects of SOCS mimetics in uveitis.

## Psoriasis

Psoriasis is a chronic, inflammatory skin condition with genetic and environmental roots, impacting about 2-3% of the global population (224) (225, 226). Often underdiagnosed, it has serious effects on quality of life and is associated with medical and psychiatric issues. Although treatments alleviate symptoms, accurate diagnosis and classification of its specific type are crucial for effective management. The most prevalent form is plaque psoriasis, ranging from mild to severe (227–229). Psoriasis’s cause involves complex interactions within the immune system, particularly T helper cells like Th1 and Th17, which lead to inflammation and thickened skin (230–234). Key pathways include JAK/STAT, NF-κB, and MAPK. IL-12 and IL-23 signaling sustains chronic inflammation by promoting cytokines (233, 235). This imbalance results in scaly plaques due to excessive keratinocyte growth (231–234).

Genetic factors significantly contribute to psoriasis. Certain genes, like HLA-C, IL23R, IL12B, and LCE3B/3C, increase susceptibility to psoriasis. Environmental triggers like infections, skin trauma, and medications exacerbate this condition in genetically predisposed individuals by activating immune responses. Targeting the JAK/STAT pathway with inhibitors, such as SOCS1-KIR, shows promise for alleviating psoriatic inflammation.

Genetic factors significantly contribute to psoriasis. Certain genes, like HLA-C, IL23R, IL12B, and LCE3B/3C, increase susceptibility to psoriasis (236, 237). Environmental factors, such as infections (streptococcal infections), trauma to the skin, and certain medications, can trigger or exacerbate psoriasis in individuals with a genetic predisposition (238, 239). These triggers can activate immune responses and further perpetuate the inflammatory processes in psoriasis. Targeting the JAK/STAT pathway with inhibitors including SOCS1-KIR, shows promise for alleviating psoriatic inflammation.

## Preclinical studies - animal models

Multiple animal models have been developed to study psoriasis and its mechanisms (spontaneous, xenograft, genetically engineered, intradermal injection of cytokines (L-23 or IL-17), and topical application of irritant imiquimod (IMQ)) resulting in skin inflammation, hyperproliferation of keratinocytes, and immune cell infiltration, mimicking key aspects of human psoriasis (240). Cytokines like IL-17, IL-23, and TNF-α are crucial in psoriasis, driving inflammation and abnormal cell proliferation. In light of this, targeting these cytokines could revolutionize the psoriasis treatment.

Tofacitinib have been investigated in several psoriasis mouse models. The topical application of Tofacitinib effectively reduces IL-22 expression in imiquimod-treated mouse ear skin (241) and IL-31 in mice with allergic dermatitis from toluene-2,4-diisocyanate (242). *In-vitro* tofacitinib has been shown to inhibit IL-23 expression in dendritic cells, suppress IL-22 expression in Th17 cells, and impede the differentiation of CD4+ T cells into Th2 cells, which are responsible for releasing IL-31 (243). Systemic delivery of tofacitinib has been shown to reduce the itch-related behaviors in the mice by controlling cytokines (IL-22, IL-23, and IL-31) and modulating epidermal nerves. Tofacitinib also influenced the expression of TSLP and IL-23, affecting both downstream and upstream signals of JAK (elevating Tslp mRNA expression and decreasing IL-23 mRNA expression). Although IL-17A expression, regulated by IL-23, remained unaffected, tofacitinib increased the density of peptidergic epidermal nerves (244). Mouse models provide valuable initial insights, but clinical trials are essential to assess JAK inhibitors’ safety and efficacy in treating human psoriasis.

## Treatment - clinical trials

Psoriasis treatment has evolved from ancient remedies to modern therapies like UV light, immunosuppressants, biologics, and personalized approaches. Ongoing research aims for better, safer options, offering hope for improved management and quality of life for patients with a specific emphasis on targeting cytokines. Tofacitinib has undergone extensive clinical trials for psoriasis treatment. In a 12-week Phase 2b trial, tofacitinib displayed notable clinical improvement and good tolerance for moderate-to-severe chronic plaque psoriasis (245, 246). Another small phase 2 clinical trials (NCT01710046) further supported its effectiveness by attenuating the pathological immune pathways in psoriasis (247). Phase 3 trials confirmed tofacitinib’s efficacy, showing it to be comparable to etanercept and superior to placebo in treating moderate-to-severe plaque psoriasis, offering a viable treatment option (248). Tofacitinib effectively treats nail psoriasis, showing continued efficacy in a two-year extension study (249). Additionally, in Asian patients with plaque psoriasis, tofacitinib was found to be more effective than placebo, with its efficacy maintained throughout a 52-week study period (250, 251). These findings support the potential of tofacitinib as a promising treatment for psoriasis, offering hope for improved management of this chronic skin disorder.

Solcitinib (GSK2586184), an oral JAK1 inhibitor, showed promise in a phase 2a trial (NCT01782664) for moderate-to-severe plaque psoriasis. In this trial, patients across different doses (100, 200, or 400 mg) experienced significant PASI 75 response rates at week 12, along with improved itch and quality of life. Adverse events were consistent across treatment groups, without a dose-related trend. Overall, Solcitinib demonstrated clinical improvement and good tolerability in treating moderate-to-severe plaque psoriasis over 12 weeks (252). Another oral JAK1 inhibitor, Abrocitinib (PF-04965842), showed efficacy and good tolerability in treating moderate-to-severe plaque psoriasis in a phase 2 trial with 59 patients (NCT02201524). Patients receiving different doses (200 or 400 mg) demonstrated improved symptoms compared to placebo. Although some abnormal laboratory test results were observed, no serious infections or bleeding events were reported. The study was terminated despite positive results (253).

Brepocitinib (PF-0670841), a dual TYK2/JAK1 inhibitor (254, 255), has undergone three clinical trials for psoriasis treatment. In a phase 1 trial (NCT02310750), it was well-tolerated up to 200 mg in healthy subjects and 100 mg in psoriasis patients, showing significant improvements in PASI scores (256). In a phase 2a trial (NCT02969018), it reduced PASI scores compared to placebo in multiple treatment groups, with continuous treatment at 30 mg once daily showing the greatest improvement (257). However, in a recent phase 2b trial (NCT03850483) using topical brepocitinib cream, it was well-tolerated but did not show significant changes compared to the vehicle in treating mild-to-moderate plaque psoriasis [288].

Ropsacitinib (PF-06826647), a dual oral TYK2/JAK2 inhibitor targeting IL-12 and IL-23 signaling (258) shows promising results in treating moderate-to-severe plaque psoriasis (259) with significant improvements seen in a phase 2b, 2020 (260). A network meta-analysis of JAK inhibitors (included eight randomized clinical trials involving tofacitinib, peficitinib, solcitinib, baricitinib, abrocitinib, and deucravacitinib) for psoriasis found tofacitinib to be the most effective, despite not being FDA-approved for psoriasis due to side effects (261). Tofacitinib and deucravacitinib showed the best responses in both efficacy and safety, supporting JAK inhibitors as a promising treatment option for moderate-to-severe plaque psoriasis. **Table 3** lists all JAK inhibitors that have been used in clinical trials for psoriasis.

**Table 3:** Psoriasis clinical trials of JAK inhibitors, chronological by the start date.

Intervention	Specificity of Inhibition	Conditions	NCT Number	Phases	Study Status	Enrollment	Start Date (M/D/Y)	Completion Date (M/D/Y)
Tofacitinib (CP-690550)	JAK3>JAK1>>(JAK2)	Psoriasis	NCT01736696	1	Completed	59	11/2002	04/2004
Tofacitinib	JAK3>JAK1>>(JAK2)	Psoriasis	NCT00678210	2	Completed	197	07/2008	08/2009
Tofacitinib	JAK3>JAK1>>(JAK2)	Psoriasis	NCT00678561	2	Completed	81	10/13/2008	07/24/2009
Tofacitinib	JAK3>JAK1>>(JAK2)	Psoriasis	NCT01186744	3	Completed	666	09/2010	01/2013
Tofacitinib	JAK3>JAK1>>(JAK2)	Psoriasis	NCT01163253	3	Terminated	2867	09/2010	06/2016
Tofacitinib Etanercept	JAK3>JAK1>>(JAK2)	Psoriasis	NCT01241591	3	Completed	1101	11/2010	01/2013
Tofacitinib	JAK3>JAK1>>(JAK2)	Psoriasis	NCT01246583	2	Completed	71	02/16/2011	11/29/2011
Tofacitinib	JAK3>JAK1>>(JAK2)	Psoriasis	NCT01276639	3	Completed	901	03/2011	04/2013
Tofacitinib	JAK3>JAK1>>(JAK2)	Psoriasis	NCT01309737	3	Completed	960	03/2011	04/2013
Tofacitinib	JAK3>JAK1>>(JAK2)	Psoriasis	NCT01519089	3	Completed	95	03/2012	01/2014
GSK2586184 (Solcitinib)	JAK1	Chronic plaque psoriasis	NCT01782664	2	Completed	68	03/01/2013	03/24/2014
Tofacitinib	JAK3>JAK1>>(JAK2)	Plaque Psoriasis	NCT01710046	2	Completed	12	03/2013	11/2013
Tofacitinib	JAK3>JAK1>>(JAK2)	Psoriasis; Vulgaris Psoriasis	NCT01831466	2	Completed	476	05/2013	09/2014
Tofacitinib	JAK3>JAK1>>(JAK2)	Psoriasis	NCT01815424	3	Completed	266	12/2013	07/2015
PF06263276 Tofacitinib Daivonex	TYK2 JAK1/3 Corticosteroid	Psoriasis Vulgaris	NCT02193815	1	Completed	15	09/2014	02/2015
PF04965842 (Abrocitinib)	JAK1	Plaque Psoriasis	NCT02201524	2	Terminated	59	11/2014	09/2015
Brepocitinib (PF-06700841)	TYK2/JAK1	Healthy; Plaque Psoriasis	NCT02310750	1	Completed	96	11/2014	02/2016
Deucravacitinib	TYK2	Psoriasis	NCT02931838	2	Completed	268	11/15/2016	11/16/2017
Brepocitinib (PF-06700841)	TYK2/JAK1	Chronic Plaque Psoriasis	NCT02969018	2	Completed	212	12/2016	03/2018
Ropsacitinib (PF-06826647)	TYK2/JAK2	Healthy Plaque Psoriasis	NCT03210961	1	Completed	109	07/14/2017	01/25/2019
Deucravacitinib Apremilast	TYK2	Psoriasis	NCT03611751	3	Completed	1020	07/26/2018	11/30/2020
Deucravacitinib	TYK2	Psoriasis	NCT03624127	3	Completed	666	08/07/2018	09/02/2020
Deucravacitinib	TYK2	Psoriasis	NCT03924427	3	Completed	74	04/10/2019	03/24/2021
Brepocitinib (PF-06700841)	TYK2/JAK1	Psoriasis	NCT03850483	2	Completed	344	04/08/2019	04/20/2021
Ropsacitinib (PF-06826647)	TYK2/JAK2	Plaque Psoriasis	NCT03895372	2	Completed	179	06/27/2019	11/26/2020
Deucravacitinib	TYK2	Psoriasis	NCT04036435	3	Active, Not Recruiting	1452	08/12/2019	07/26/2026
Deucravacitinib	TYK2	Psoriasis	NCT04167462	3	Completed	220	11/25/2019	01/07/2022
Deucravacitinib	TYK2	Plaque Psoriasis	NCT04772079	3	Recruiting	153	03/23/2021	09/09/2031
Deucravacitinib	TYK2	Psoriasis	NCT05478499	4	Recruiting	150	10/06/2022	10/07/2024
Adalimumab, Secukinumab, Ixekizumab, Guselkumab, JAK inhibitors, methotrexate		Psoriasis	NCT05503875	N/A	Recruiting	100	01/01/2023	12/31/2027
Deucravacitinib	TYK2	Psoriasis	NCT05701995	4	Recruiting	250	01/31/2023	01/29/2025
Deucravacitinib	TYK2	Psoriasis Vulgaris	NCT05858645	4	Not Yet Recruiting	25	07/2023	06/2026

## SOCS mimetics in psoriasis

In 2012, Doti et al. introduced PS-5, a new peptide inhibitor of JAK2, which is a mimetic of the kinase-inhibitory region of SOCS1 (262). This peptide (DTC(Acm)RQTFRSH) has a distinct amino acid composition and length compared to SOCS1-KIR. Specific amino acid substitutions in PS-5, involving phenylalanine and arginine residues, led to improved JAK2 binding by establishing enhanced electrostatic interactions with the negative phosphate moiety on Y1007. PS-5, containing a nonnatural residue (Cys(Acm)), exhibited greater protease stability and effectively inhibited STAT1 phosphorylation, leading to reduced interferon regulatory factor-1 (IRF-1) expression through binding-assay screening (262). In a 2013 study, Madonna et al. explored the therapeutic potential of PS-5 in managing IFN-γ-mediated skin pathogenesis. The research compared PS-5 with the full kinase inhibitory region of SOCS1 protein for its ability to suppress inflammatory gene expression in IFN-γ-treated human keratinocytes. PS-5 mimetic showed comparable effectiveness to the kinase inhibitory region peptide, inhibiting Jak, IFN-γRα, and STAT1 phosphorylation, along with the expression of ICAM-1, HLA-DR, CXCL10, and CCL2 (194). These findings highlight PS-5 as a promising novel therapeutic strategy for IFN-γ-induced skin pathogenesis, as depicted in **Figure 6**.

A distinct SOCS3 mimetic peptide, referred to as the KIRESS peptide (sequence: ^22^LKTFSSKSEYQLVVNAVRKLQESG^45^), was synthesized and assessed *in vitro*. This peptide spans both the KIR (Kinase Inhibitory Region) and ESS (Extended SH2-Substrate Binding) regions (195). KIRESS peptide was shown to inhibit the IL-22 signaling pathway by regulating the STAT3 and ERK 1/2 signaling, along with suppression of STAT3 expression in keratinocytes. *In-vivo* KIRESS peptide effectively suppressed tumor growth and increased STAT3 activation in athymic nude mice harboring squamous cell carcinoma (SCC) xenografts (196). While, these results are encouraging, the specificity and efficacy of these mimetic peptides in an *in-vivo* setting requires further investigation, necessitating additional studies.

## Autoimmune encephalitis

Autoimmune encephalitis (AE) is a rare, severe neurological disorder involving brain inflammation from an autoimmune response against synaptic antigens (263). The immune system mistakenly targets and attacks healthy brain tissue, leading to a range of neurological symptoms. A list of commonly reported AE autoantibodies includes: N-methyl-D-aspartate receptor (NMDAR) (264), anti-leucine-rich glioma-inactivated protein 1 (LGI1) (265), contactin associated protein-like 2 receptors (CASPR2) (266), gamma aminobutyric acid (GABA) (267, 268); alpha-amino-3-hydroxy-5-methyl-4-isoxazolepropionic acid (AMPA) (269), dipeptidyl-peptidase-like protein-6 (DPPX) (270), and myelin oligodendrocyte glycoprotein (MOG) (271). Currently, NMDAR encephalitis is the most common and well-studied subtype of AE (272).

AE presents cognitive impairments, seizures, memory problems, behavioral changes, movement disorders, and psychiatric symptoms (273). It require careful diagnosis and appropriate treatment, often involving immunosuppressive therapies (274). Antibodies in AE are intrinsically pathogenic. They target synaptic proteins and induce conformational changes leading to widespread inflammation (275). A prior infection that caused inflammation and neurological symptoms is a common precursor to AE. Herpes simplex virus encephalitis (HSVE) has been shown to trigger immune response causing NMDAR encephalitis (276). Moreover, the human leukocyte antigen (HLA) is linked to the production of antibodies that trigger autoimmune responses (277). Up to the present time, a considerable number of comparable syndromes, often termed AE, have been discovered. In neuron cultures, pathogenic antibody effects have been shown for various AE types. These effects include receptor blocking (GABABR), receptor cross-linking and internalization (NMDAR) (278, 279), and disruption of protein-protein interactions (LGI1) (280). Yet, the lack of suitable animal models continues to restrict our comprehension and development of novel therapies. Anti‐NMDAR encephalitis is a prevalent (281) and best studied subtype of AE, for which a few mouse models were developed (75, 282, 283). Studies have explored the pathogenic effects of patient-derived or a human recombinant antibody in passive-transfer animal models. Planaguma et al. demonstrated that infusing CSF from anti-NMDAR encephalitis patients altered memory and behavior in mice. The antibodies disrupted NMDAR interaction with the ephrin-B2 receptor, leading to receptor internalization and impaired synaptic plasticity, memory, anhedonia, and depressive behavior. After the antibody infusion was terminated, these changes gradually resolved (75). Other studies indicated that passive NMDAR antibody transfer from patients to mice could induce seizures (283) and psychotic behavior in mice (282). These findings demonstrate the potential of AE patient autoantibodies for creating precise mouse models, providing insights across multiple levels, including cellular, synaptic, and neural networks, and facilitating novel therapy testing. A postinfectious autoimmune encephalitis mouse model developed by performing multiple intranasal infections with live group A *Streptococcus* (GAS) shows Th17 cells migration from the nose into the brain, resulting in the disruption of the blood-brain barrier (BBB) and the inflow of autoantibodies into the CNS (284). Later, the same group highlighted the essential role of Th17 lymphocytes in enabling selective CNS autoantibody entry, microglial activation, and neural circuit impairment in postinfectious AE. Mice lacking Th17 cells exhibited reduced BBB leakage, microglial activation, CNS antibody infiltration, and partial olfactory function restoration (285). In AE, the JAK-STAT pathway can become dysregulated, leading to abnormal immune responses and inflammation within the central nervous system (CNS). Tofacitinib’s ability to cross the blood-brain barrier and modulate cytokine receptors positions it as a potential therapy for AE and refractory AE, offering hope for more targeted treatments (286, 287).

## Preclinical studies - animal models

Autoimmune Encephalitis (AE) is predominantly a human condition and is less frequently observed in mice. Animal models that mimic aspects of AE have been instrumental in providing direct evidence of the pathogenicity of autoantibodies. For instance, researchers have created these models by transferring cerebrospinal fluid (CSF) or immunoglobulins from patients with anti-NMDA receptor encephalitis to mice (283). These studies have shed light on a critical process: the continuous production of autoantibodies by self-reactive B cells. Two major pathogenic pathways have been documented in autoimmune encephalitis: one involving the selective and reversible reduction of NMDA receptor surface density and synaptic localization upon exposure to autoantibodies from anti-NMDAR encephalitis patients (279, 288), and the other pathway involves complement activation, demonstrated in CASPR2 antibody-associated encephalitis cases (289). Despite extensive research and increasing clinical insights, a notable proportion of patients still do not benefit from existing treatments. A recent study reported a translational rodent model of NMDARE (n-methyl-D-aspartic acid receptor (NMDR) encephalitis), using active immunization and offers a valuable tool for delving into the pathophysiology of AE (290). This development holds promise for advancing the diagnosis and treatment of this debilitating neuropsychiatric condition with a relatively rapid onset of the phenotype, enabling in-depth investigations into its pathophysiology. Additionally, the NMDARE mouse model has the potential to serve as an effective translational platform for pre-clinical testing of both existing and future therapeutic interventions.

## Clinical trials

Autoimmune encephalitis (AE) is a rare and serious medical condition that involves inflammation of the brain (51, 291), and treatment options are based on autoimmune disease management principles (274, 277, 292). Immunotherapy, such as corticosteroids, intravenous immunoglobulins, and plasmapheresis, is the first-line therapy, rituximab and cyclophosphamide (second line therapy) and other immunosuppressive drugs used when needed (293). Controlled clinical trials for AE are lacking due to the rarity of the disease (294–296). However, ongoing trials, such as one evaluating bortezomib (NCT03993262), a proteasome inhibitor, in severe AE, hold promise for establishing guidelines and advancing to larger phase III trials (295). In refractory AE, blood-brain barrier penetrating novel immunotherapies are crucial (297). Tofacitinib, a JAK3/1 inhibitor, has shown potential in treating refractory immune-mediated diseases, and recent studies suggest it could be a promising option for some AE patients (298). However, further research with a larger patient group is needed to fully understand its effectiveness in AE. As tofacitinib penetrates the BBB (242, 299) it has the potential to be effective in the CNS autoimmune disorders, however further analysis of much larger group of patients is required to make a conclusion for the use of tofacitinib in AE. Despite progress in understanding AE (300), the exact pathological mechanisms remain unclear, and therapeutic options are currently limited.

## Systemic lupus erythematosus

Systemic lupus erythematosus (SLE) is a complex autoimmune disorder with a wide-ranging impact on the body. Common manifestations include joint pain, skin rashes (especially the characteristic “butterfly” rash on the face), fatigue, and fever. SLE is more frequent in women. The precise mechanism of SLE pathogenesis is still unknown. SLE highlights the intricate interplay between genetics, environmental factors, and immune dysregulation. Cytokines are crucial in the development of SLE’s pathophysiology (301, 302). Cytokines, including interleukin-10 (IL-10) and B-cell activating factor (BAFF), have a pivotal role in the activation, survival, and differentiation of B cells. Additionally, cytokines such as interleukin-2 (IL-2) and interleukin-21 (IL-21) impact T-cell function and differentiation. Elevated levels of specific cytokines such as interleukin-6 (IL-6), interleukin-17 (IL-17), and tumor necrosis factor-alpha (TNF-alpha) are a common occurrence in individuals with SLE (303, 304). Elevated type I interferons (IFNs) correlate strongly with active SLE, driving autoantibody production, immune complex formation, and tissue damage. JAK1 and TYK2, downstream signals of IFN, show significant associations with SLE, especially TYK2 polymorphism (305). [357]. In patients with SLE, there is a substantial increase in the expression of CXCR4, a crucial receptor involved in immune regulation (306, 307). This heightened expression is intricately associated with the activation of the JAK/STAT pathway, fostering the infiltration of immune cells into the kidneys, thereby aggravating the progression of the disease. These findings suggest the potential use of JAK inhibitors for treating lupus (308).

## Preclinical studies - animal models

Over time, a diverse range of murine models for lupus has been created and utilized, each with its own limitations, contributing to our understanding of systemic lupus erythematosus (SLE) (309). Among these models, the MRL/lpr mice distinguish themselves by their exceptional capacity to generate an extensive spectrum of lupus-associated autoantibodies (ANA, anti-dsDNA, anti-Sm, anti-Ro, and anti-La) alongside exhibiting features such as arthritis, cerebritis, skin rash, and vasculitis (310).

Promising outcomes in mouse models have been demonstrated by various JAK inhibitors. Ruxolitinib reduced severe skin lesions in MRL/lpr mice, but its effect on other lupus manifestations remains unclear, requiring further research (311). Tofacitinib demonstrated reduced disease activity, nephritis, and autoantibody titer in MRL/lpr and NZB/NZWF1 mice, suggesting potential as a therapy for SLE (312–315). Baricitinib, a selective JAK1/2 inhibitor, significantly suppressed lupus-like symptoms and restored disrupted podocyte structures (316). Another potential therapy, Deucravacitinib (BMS-986165), showed reduced IFN expression in SLE patient cells and decreased type I IFN-regulated gene expression in NZB/W mice (317). These results underscore the promise of JAK inhibitors as innovative solutions for SLE, yet additional research and clinical trials are required in human patients.

## Treatment of SLE patient – clinical trials

Immunosuppressive medications and glucocorticoids are the two most common current SLE treatments, marked by notable side effects and limited efficacy. Under these circumstances, SLE still has a high rate of morbidity and mortality. Identification of bioactive agents has positioned the JAK/STAT pathway as a more suitable contender for SLE’s pathogenesis.

The first JAK1 inhibitor, GSK2586184 (Solcitinib), was assessed for its effectiveness, safety, and tolerability in adults with SLE (ClinicalTrials.gov NCT01777256). However, the study revealed various safety issues in participants (318, 319). Consequently, the trial was deemed futile, and recruitment was stopped after 50 patients. Based on the safety and efficacy statistics from the study, further research on GSK2586184 in SLE patients is not recommended. Tofacitinib (CP-690550) was then evaluated and showed promise in treating SLE [380]. Another recent randomized, double-blind, placebo-controlled study (NCT02535689) demonstrated that tofacitinib is safe and well-tolerated in patients with mild-to-moderate SLE. Tofacitinib reduced IFN type I signature, improved lipid profile, restored endothelial function, and enhanced cardiometabolic and immunologic parameters associated with premature atherosclerosis. Additionally, the drug’s protective benefits were more pronounced in individuals with the STAT4 risk allele, which is linked to more severe SLE and an increased risk of cardiovascular (CV) events (320). A case report evaluating the efficacy of tofacitinib in three patients with recalcitrant cutaneous lupus reported favorable outcomes, showing significant improvement (321). Ongoing and approved trials of tofacitinib in SLE aim to gather more data on targeting the JAK pathway’s effectiveness, with potential implications for cardiovascular function across autoimmune disorders and the broader population.

Baricitinib selectively inhibits JAK1 and JAK3 subtypes. A double-blind placebo-controlled study in 314 lupus patients with skin and joint manifestations (NCT02708095) showed positive results. After 24 weeks of baricitinib (4 mg) treatment, 67% of patients achieved resolution of arthritis or rash (322), along with reduced expression of key lupus-related cytokines, improved SLE disease activity, anti-dsDNA antibody levels, and diminished swollen and sore joints (323). It showed potential in treating cutaneous and articular involvements (322, 324, 325), but its efficacy in lupus nephritis is still being investigated in a phase 2/3 trial (NCT05686746). Phase III trials for moderate to severe SLE adult patients (NCT03616964 & NCT03616912) had inconclusive outcomes. A follow-up study for long-term safety (NCT03843125) was terminated due to insufficient evidence of positive benefit. Further pre-clinical data are needed to better understand baricitinib potential efficacy and mechanism in treating lupus-related phenotypes.

Filgotinib (GLPG0634) treatment, a selective JAK1 inhibitor, showed disappointing outcomes in a cutaneous lupus erythematosus trial, failing to significantly improve CLASI scores (326). Its use in lupus membranous nephropathy yielded unsatisfactory results with only limited conclusions due to a small number of participants (327). Presently, no planned trials involve filgotinib in SLE patients. In contrast, Upadacitinib (ABT-494), a second-generation selective JAK1 inhibitor, has been approved for conditions like rheumatoid arthritis (328), psoriatic arthritis (329), and atopic dermatitis. Limited data on its efficacy in SLE exists, with only one case report showing resolution of accelerated nodulosis (330). Phase II trials (NCT04451772) have been completed to evaluate the safety and efficacy of upadacitinib in SLE, and phase III (NCT05843643) trials anticipated to commence soon [www.clinicaltrials.gov last accessed June 6, 2023].

In healthy volunteers, Deucravacitinib demonstrates favorable pharmacokinetics and safety traits (331) and is now FDA approved to manage moderate-to-severe plaque psoriasis (332). A recent phase II trial in adult SLE patients (NCT03252587) reported positive outcomes, with a higher SLE Responder Index 4 (SRI-4) response compared to placebo (333). The trial met all secondary endpoints, indicating deucravacitinib efficacy and acceptable safety. The ongoing phase 3 trials, (NCT05617677 & NCT05620407) are assessing its efficacy and safety. A study (NCT03920267) is also assessing its long-term safety and efficacy in SLE.

JAK inhibitors of the first generation address multiple JAK isoforms, yielding diverse impacts yet also triggering a spectrum of side effects (330). Although SLE clinical trials have demonstrated potential benefits, none of the JAK-STAT inhibitors listed in **Table 4** have yet garnered approval for use in SLE clinical practice. Tofacitinib and baricitinib have shown the most promising outcomes so far. Nonetheless, while their safety and effectiveness profiles present encouraging data, further clinical trials are imperative for future validation.

**Table 4:** SLE clinical trials of JAK inhibitors, chronological by the start date.

Intervention	Specificity of Inhibition	Conditions	NCT Number	Phase	Study Status	Enrollment	Start Date (M/D/Y)	Completion Date (M/D/Y)
GSK2586184 (Solcitinib)	JAK1	SLE	NCT01777256	2	Terminated	51	3/1/2013	3/31/2014
Tofacitinib (Tasocitinib or CP-690550)	JAK3>JAK1>>(JAK2)	SLE	NCT02535689	1	Completed	34	8/28/2015	4/26/2018
Baricitinib (INCB028050 or LY3009104)	JAK1/JAK2	SLE	NCT02708095	2	Completed	314	3/24/2016	11/9/2017
Tofacitinib	JAK3>JAK1>>(JAK2)	SLE; Discoid Lupus Erythematosus	NCT03159936	Early | 1	Terminated	5	4/3/2017	6/10/2020
Filgotinib	JAK1	Cutaneous Lupus Erythematosus	NCT03134222	2	Completed	47	4/28/2017	6/9//2020
Tofacitinib	JAK3>JAK1>>(JAK2)	SLE; Cutaneous Lupus	NCT03288324	1 | 2	Completed	13	8/23/2017	12/1/2022
Filgotinib & Lanraplenib	JAK1	Lupus Membranous Nephropathy (LMN)	NCT03285711	2	Completed	9	9/18/2017	5/18/2020
Deucravacitinib (BMS-986165)	TYK2	SLE	NCT03252587	2	Completed	363	9/21/2017	10/28/2021
Baricitinib	JAK1/JAK2	SLE	NCT03616964	3	Completed	778	8/2/2018	10/20/2021
Baricitinib	JAK1/JAK2	SLE	NCT03616912	3	Terminated	830	8/2/2018	3/9/2022
Deucravacitinib	TYK2	SLE	NCT03920267	2	Active Not Recruiting	261	3/26/2019	3/31/2025
Elsubrutinib & Upadacitinib (alone or in combination (ABBV-599))	BTK JAK1 BTK & JAK1	SLE	NCT03978520	2	Completed	341	7/25/2019	7/14/2022
Baricitinib	JAK1/JAK2	SLE	NCT03843125	3	Terminated	1147	9/8/2019	4/1/2022
Elsubrutinib & Upadacitinib (alone or in combination (ABBV-599))	BTK JAK1 BTK & JAK1	SLE	NCT04451772	2	Active Not Recruiting	260	7/27/2020	12/28/2023
Baricitinib	JAK1/JAK2	Lupus or SLE Nephritis	NCT05686746	2 | 3	Recruiting	80	6/1/2022	9/1/2023
Tofacitinib	JAK3>JAK1>>(JAK2)	SLE; Cutaneous Lupus	NCT05048238	1	Recruiting	10	9/30/2022	12/2023
Deucravacitinib	TYK2	SLE	NCT05617677	3	Recruiting	490	1/12/2023	12/17/2027
Deucravacitinib	TYK2	SLE	NCT05620407	3	Recruiting	490	1/12/2023	12/17/2027
Upadacitinib (ABT-494)	JAK1>>(JAK2)	SLE	NCT05843643	3	Not Yet Recruiting	1,000	6/30/2023	10/31/2027

## SOCS mimetics in SLE

Cytokine imbalance and the diminished SOCS1 expression both hold significant roles in the advancement of SLE. Restoring or enhancing SOCS1 expression or function could potentially modulate cytokine signaling, reduce inflammation, and attenuate the progression of SLE. SOCS1 mimetics could be a safe alternative.

Reduced SOCS1 expression in individuals with systemic lupus erythematosus (SLE) and in lupus mouse models is established (334–336). This decline in SOCS1 expression exhibits a negative correlation with the extent of inflammation. The decreased expression of SOCS1 contributes to the dysregulated cytokine signaling and excessive immune activation seen in SLE. Hematologic abnormalities, including abnormal blood cell counts, and the generation of autoantibodies are influenced by SOCS1 insufficiency. Aberrations in SOCS1 also contribute to lupus nephritis, a grave complication of SLE, influencing organ performance in regions like the skin, central nervous system, heart, and kidneys (335, 337).

Research by Sukka-Ganesh and Larkin was aimed to identify a therapeutic potential to target SOCS1 in the treatment of SLE by using mice and studying SLE patients and clinical samples. The study findings revealed that after 48 hrs. of stimulation with anti-CD3, LPS, or IFN-γ, SOCS1 and SOCS3 expression peaked in murine splenic samples. Furthermore, the peripheral blood mononuclear cells (PBMCs) from SLE patients exhibited notably lower levels of both SOCS1 and SOCS3 mRNA and protein compared to control subjects. The study also demonstrated that reduced SOCS1 levels in SLE patients were associated with increased inflammatory markers and upregulated expression of major histocompatibility complex (MHC) class II molecules. Moreover, patients receiving steroid exhibited elevated levels of SOCS1 compared to untreated, and the human PBMCs treated with steroid showed dose- and time-dependent upregulation of SOCS1 mRNA (336).

The therapeutic potential of SOCS1-KIR in modulating lupus-associated pathologies has been recently evaluated in Fas-deficient MRL/lpr mice. The application of SOCS1-KIR led to diminished skin lesion severity, lowered production of autoantibodies, and moderate enhancements in kidney pathology. At the cellular level, the introduction of SOCS1-KIR through peritoneal administration augmented the expression of Foxp3 in overall splenic and follicular regulatory T cells, lowered the ratio of effector memory to naive T lymphocytes in both CD4+ and CD8+ cell populations, and diminished the occurrence of germinal center B cells. These observations suggest that SOCS1-KIR treatment triggers changes in lymphocyte dynamics, hinting at the therapeutic potential of peptide administration for alleviating SLE-related pathology (193) (**Figure 6**).

## FDA and EMA approved JAK inhibitors

**Figure 7:**
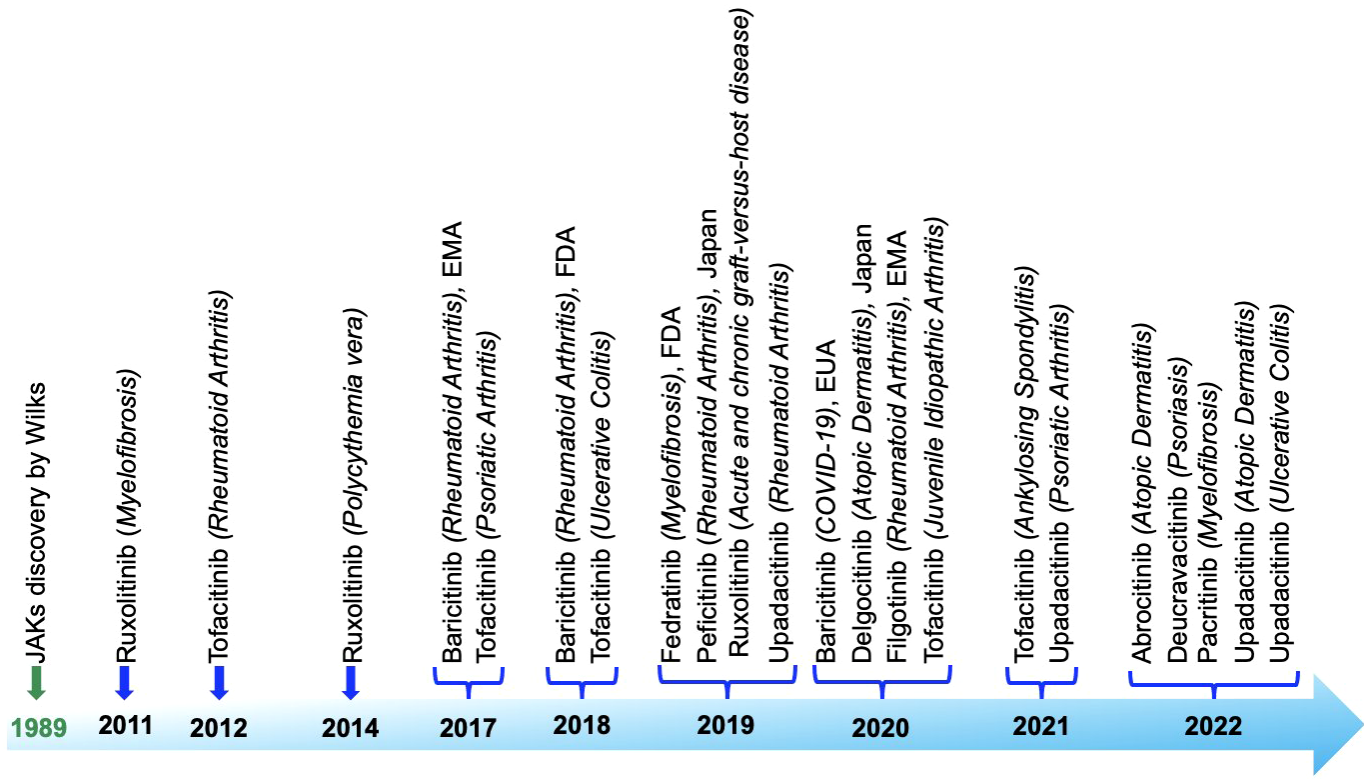
Roadmap detailing the discovery and approval of Janus kinase inhibitors, highlighting the key milestones and stages in the process.

JAK inhibitors, also known as Jakinibs, belong to a class of small-molecule medications. These inhibitors hold promise in treating autoimmune conditions and have proven effectiveness against inflammatory diseases such as rheumatoid arthritis and psoriasis, among others. Administered orally, they initially targeted multiple JAK enzymes, but newer iterations are more discerning. Despite their swift action, drawbacks exist, encompassing side effects (gastrointestinal problems, liver irregularities, anemia, changes in blood lipids), high expense, and heightened susceptibility to infections and malignancies. Long-term safety data is still being collected. **Figure 7** roadmap showcases the expanding use of JAK inhibitors in treating distinct autoimmune and inflammatory disorders, underlining their growing significance in therapeutic interventions that have obtained approval from both the European Medicines Agency (EMA) and the United States Food and Drug Administration (FDA) in chronological order based on first approval: Ruxolitinib (338–340), Tofacitinib (341–344), Baricitinib (343, 345, 346), Fedratinib (347), Peficitinib (348), Upadacitinib (328, 329, 349, 350), Delgocitinib (351), Filgotinib (352), Abrocitinib (353), Deucravacitinib (332), Pacritinib (354).

## Conclusions

The field of autoimmune research is constantly progressing. Currently, there are 2,254 planned studies focused on autoimmune diseases (https://clinicaltrials.gov). Examining and refining Jakinibs through development and testing has illuminated the specific functions that different JAKs play in various human diseases. SOCS-KIR peptides show promise as cutting-edge therapeutic options for autoimmune disorders, thanks to their small size, stability, and low immunogenicity, which make them favorable candidates for safe therapeutic development. SOCS1-KIR, when internalized by cells, has shown strong therapeutic potential in EAE, autoimmune uveitis, psoriasis, and diabetes models. In contrast, SOCS1 antagonist (pJAK2 (1001–1013)) has been shown to enhance immune responses against various viruses. SOCS mimetics and antagonists hold promise as potential therapeutics for regulating the immune system in both negative and positive ways. However, it remains imperative to conduct additional research and clinical trials to gain a comprehensive understanding of their mechanisms of action, safety, and effectiveness across various autoimmune conditions before considering their widespread utilization in clinical practice. Scientists and the medical community are diligently striving to devise and apply inventive methodologies aimed at targeting JAK-STAT pathways and creating mimetics that target SOCS. SOCS1 mimetics hold promise for treating disorders associated with excess inflammation or SOCS1 deficiency upon fully established safety studies.

## Author contributions

RP: Conceptualization, Methodology, Writing – original draft, Writing – review & editing. MB: Conceptualization, Methodology, Writing – review & editing. HH: Conceptualization, Methodology, Supervision, Writing – review & editing.
